# A Roadmap for the Application of Convalescent Plasma and Hyperimmune Globulins in Emerging Viral Outbreaks

**DOI:** 10.1155/tswj/9976167

**Published:** 2025-07-03

**Authors:** Aliasghar Rahimian, Hooman Askari, Ali Nabati, Mahdi Aminian

**Affiliations:** Department of Clinical Biochemistry, Tehran University of Medical Sciences, Tehran, Tehran Province, Iran

**Keywords:** convalescent plasma, emerging viruses, hyperimmune globulin, viral outbreaks

## Abstract

The outbreak of COVID-19 took the world by surprise and dealt a serious blow to the global economy. Even today, few drugs against SARS-CoV-2 infection have been proven useful, and repurposing existing antiviral therapies remains a major research area in the treatment of the disease. During previous viral outbreaks, therapies using convalescent plasma and related products have repeatedly been used as empirical approaches. Rapid preparation of convalescent plasma and hyperimmune globulins (hIVIGs) makes these two treatment options useful in dealing with outbreaks of emerging diseases. The current study presents a systematic roadmap concerning the guidelines, criteria, and regulations involved in plasma transfusion and the preparation of hIVIGs applicable to possible future viral outbreaks.

## 1. Introduction

Outbreaks of emerging viral infections are seen around the world at an increasing rate [1]. Researchers have described several factors as to why an apparent increase in the occurrence of such infections is observed [2–4]. Regardless of the underlying reasons for this phenomenon, precautionary measures should be taken to avoid the disorderliness caused by these outbreaks. SARS-CoV-2 and Ebola virus disease (EVD) are two of the most well-known examples of such events, during which many local and international healthcare systems were overwhelmed, and with hindsight, not all policies implemented were consistent and appropriate [5–10]. The EVD outbreaks from 2014 to 2016 occurred under rather different circumstances. EVD outbreaks have been occurring continuously since 1976, when the first subtype, Zaire, was discovered [11]. Despite this, no effective treatments or vaccines were available when a major outbreak occurred in Guinea in 2013. This was possibly due to a lack of incentive and an underestimation of the EVD threat [4, 12]. Unlike respiratory viruses, EVD is not transmitted through the air, and some kind of direct or indirect contact with bodily fluids is necessary. In any event, a situation similar to the SARS-CoV-2 outbreak was experienced in countries most affected by the disease. It was ultimately possible to contain the disease by isolating identified cases, increasing public awareness, and using protective equipment. As a possible treatment option for both SARS-CoV-2 and EVD, convalescent plasma transfusion has been proposed as a practical investigational approach [13]. The same was true with the outbreak of avian influenza, where convalescent plasma obtained from recovering patients was considered a last resort to improve the survival rate of those infected [14–20]. This is not surprising as plasma transfusion is a well-established technique, and its use as a therapeutic approach in viral outbreaks dates back as early as 1918, when it was used for the first time in the treatment of Spanish flu outbreaks [15, 21]. Regarding the SARS-CoV-2 outbreak, convalescent plasma was the subject of clinical trials for severely ill patients undergoing intensive care. Although WHO strongly advises against the use of convalescent plasma in nonsevere COVID-19 patients based on the findings of clinical trials [7], the use of convalescent plasma and its related products was extremely common at the beginning of the COVID-19 pandemic [22]. It seems most rational to assume that convalescent plasma and neutralizing antibodies are to be used again in future situations involving the outbreak of a new viruses. Here, we explain a roadmap for the rapid establishment of two convalescent plasma–based therapies: plasma transfusion and purified hyperimmune globulins (hIVIGs). Our review discusses the basic necessities for the urgent initiation of plasma transfusions from recovered patients to critically ill patients. In addition, it outlines the regulatory requirements for the purification and manufacture of hIVIG from convalescent plasma against an emerging viral outbreak.  Table 1 summarizes the benefits and drawbacks of using convalescent plasma and hIVIG in the treatment of patients with emerging viral infections.

## 2. Donor Selection Process

### 2.1. An Overview of the General and Specific Criteria for Selecting Blood Donors

Transfusion of blood products is a routine medical and healthcare practice with well-established protocols, especially for donor selection; therefore, there is no need for the development of new regulations. The general donor eligibility criteria are extensively discussed by both Red Cross and WHO [23, 24]. In addition, the USP states that the minimal donor selection criteria should be in accordance with the federal code of regulations (CFRs), 21 CFR § 640.31. A detailed discussion of criteria regarding noncommunicable diseases, patient medical history, and conditions can be found in the WHO guideline and is beyond the scope of this article [23–28].

During previous viral outbreaks, health authorities established donor selection guidelines. In response to the outbreak of Ebola, WHO published interim guidance accompanied by a set of recommendations for the assessment of donors as well as pre- and postdonation testing [20]. Furthermore, the guidance stipulates that only recovered EVD patients who have been clinically asymptomatic and have tested negative twice in RNA testing (with at least 48-h intervals) should be considered eligible for donation. It is also stated in the guidance that the criteria for selecting donors (specifically age) may be relaxed in situations where the risk of transfusion is lower than the risk of receiving no treatment at all for both the donor and the recipient [20]. The FDA candidate selection criteria for investigational convalescent plasma in the treatment of COVID-19 patients can also be exploited in establishing a general guideline for viral outbreaks [29]. The guideline suggests that convalescent plasma may be collected from individuals who meet three specific criteria when used under Investigational New Drug (IND) regulations: (1) evidence of SARS-CoV-2 infection documented by laboratory testing, (2) complete remission of symptoms for at least 10 days before plasma donation, and (3) female donors with a prior pregnancy history qualify only if they have tested negative for HLA antibodies. It should be noted that the FDA states negative test results for COVID-19 are not necessary for donor qualification, and apparent remission of symptoms is enough. This is clearly contradictory to what was proposed in the guideline published for convalescent plasma therapy for EVD patients, as it stated that individuals should be negative for viral RNA with a minimum of 48-hours interval. In addition, the guideline states that convalescent plasma should be tested for anti-SARS-CoV-2 antibodies. Neutralizing antibodies are expected to be in acceptable titers 14 days after patients have been discharged from treatment centers [29, 30]. The use of convalescent plasma for COVID-19 under an Emergency Use Authorization (EUA) is only justified in cases where the patient suffers from a defective immune system (caused by taking immunosuppressive drugs or a certain disease) [31]. The criteria are similar to those required under an IND with an exception; the guideline indicates that donors who have received any investigational, FDA-approved vaccines, or monoclonal antibodies can be donors only with certain conditions. According to the FDA, this last criterion is set to ensure that the antibodies present in the plasma are generated against SARS-CoV-2. Neutralizing antibodies are defined as antibodies capable of binding to a virus and preventing it from infecting its host cells [32]. Evidently, these antibodies are responsible for the humoral immunity generated against viral diseases after receiving vaccines or recovering from an infection [33]. Measurement of virus-neutralizing antibodies is of great importance for purposes such as assessment of vaccine efficacy, immunity against the virus in individuals recovered from an infection, and the quality of convalescent plasma obtained for treatment [32]. The gold standard method for measuring virus-neutralizing antibodies is the plaque-reduction neutralization test (PRNT). The method requires incubation of the live virus with the patient (donor) sera and then adding the mixture to a eukaryote cell layer. Clearly, the handling and management of live viruses require special precautions and well-equipped laboratories. In the cases of SARS-CoV-2 and Ebola virus outbreaks, PRNT could be done only in BSL-3 or BSL-4 laboratories, which makes screening of large numbers of samples impossible through this method [33–36]. Therefore, alternate methods are routinely employed to measure the level of neutralizing antibodies. FRNT, or focus reduction neutralization test, employs antibodies generated against specific antigens on the surface of the virus to perform immunostaining after the treatment of the eukaryote cells with the live virus incubated with serum containing neutralizing antibodies. The technique reduces the time required for performing a measurement of neutralizing antibodies; however, it still requires handling the native virus and special safety precautions. In order to eliminate the risks associated with the use of pathogenic viruses, reporter virus particles are commonly employed in various types of assays. The reporter viruses are replication-incompetent and typically consist of a viral core from a retrovirus and surface protein/proteins of the target virus. A reporter gene such as luciferase or GFP is also incorporated in the reporter virus to provide a straightforward means of detection [33, 37–39]. The main limitation of this kind of assay is its inability to measure neutralizing antibodies generated against the viral core [39]. Less accurate estimations of neutralizing antibodies might be obtained from ELISA and lateral flow immunoassays [33]. Looking back at previous viral outbreaks, setting up a reliable neutralizing antibody test during an emergency would at least provide some insight into the efficacy of transfusion and a basis for devising preliminary selection criteria. It should be noted that in very emergency situations where overwhelming patients with active viruses are present, serum assessment for neutralizing antibody titer could be skipped provisionally for convalescent plasma therapy. In cases involving an emerging viral outbreak, appropriate donor selection criteria should be established. The below criteria are recommended (Table 2 lists the major guidelines for donor selection):

1. The donor should meet the general blood donation criteria established by international or local health authorities or blood transfusion services [23, 24, 29].
2. To exclude the possibility of transmission of active viruses, the donor blood must be proven to be free of any active viruses by reliable molecular diagnostic testing (at least twice tested by real-time PCR).
3. The donor should contain an acceptable level of neutralizing antibodies. A consensus has not been reached on the anti-SARS-CoV-2 neutralizing antibody titer threshold for plasma donation. Nonetheless, a recent FDA guidance recommends a neutralizing antibody titer greater than 1:160 for investigational plasma therapy for COVID-19 [29]. In the case of Ebola, a cut-off level of more than 1:160 was recommended for plasma donor selection.

### 2.2. Diagnostic Methods for Detecting an Active Viral Infection

As it was noted, a reliable diagnostic test should be employed to avoid viral infection transmission by plasma transfusion [40]. The gold standard method for identifying and isolating viruses from clinical samples is to culture them in specified cell lines [40]. However, being both time- and resource-consuming, viral culture is rarely recommended in guidelines for viral infection diagnosis. Today, nucleic acid amplification techniques (NATs) seem to work best as they offer a great deal of sensitivity while requiring minimal resources [25].

Regarding the outbreak of Ebola, WHO suggested that donor blood be tested twice for Ebola RNA by molecular techniques. Additionally, it was recommended that two separate blood samples be collected at least 48 h apart, and both samples must be negative for Ebola RNA in order to qualify the donors.

The USP states that testing for viruses with a relatively low load during the asymptomatic stage of the disease, such as SARS-CoV, is unnecessary. Also, it is critical that all diagnostic tests meet the Clinical and Laboratory Standards Institute (CLSI) and other relevant guidelines [25]. At the beginning of the COVID-19 outbreak, WHO suggested that the interim report published in response to the outbreak of Ebola be taken into consideration [41]. Consequently, most of the trials performed at the time required the donors to be negative for the presence of COVID-19 RNA in their blood sample in two separate PCR tests [42]. Later on, the WHO deemed the PCR testing unnecessary and declared that the absence of symptoms alone would be sufficient to qualify the plasma donors [7]. Be that as it may, it seems that assessing recovering donors by molecular testing targeting the virus's genetic material would be the best approach to ensure the safety of convalescent plasma when tackling an emerging viral infection.

## 3. Plasma Therapy (Convalescent Plasma Transfusion)

### 3.1. An Overview of General Criteria Required for Plasma Transfusion

Plasma transfusion is performed in a range of clinical situations, often to increase the blood volume in cases of severe blood loss and as a homeostatic agent. There are guidelines established by health authorities that define the appropriate courses of action in the administration of plasma transfusion, but these will not be discussed here. In many cases, it is the responsibility of the health practitioners to determine whether plasma transfusion should be done or not. While for most situations the guidelines exist to help these practitioners make the right orders, in cases involving the outbreak of a new pathogen, the decision is much more arbitrary rather than evidence-based. Nevertheless, inclusion and exclusion criteria must be settled under the supervision of healthcare authorities. COVID-19 and EVD outbreaks are two of the most well-known situations where convalescent plasma (plasma transfusion from recovered patients) was used as an empirical treatment. Reviewing the main points of the official documents related to these outbreaks might help us to get some insight as to how the patients should be selected for receiving convalescent plasma.

In addition, it is stated that venous blood samples should be taken from the patient and collected in tubes with or without anticoagulant (to obtain plasma and serum) in volumes of 5 mL. The plasma sample is used for ABO and RhD blood typing, while the serum sample is used to measure the level of viral load before receiving the treatment (establishing a baseline). It is also stated that another serum sample should be taken a day after the transfusion to measure the viral load after the treatment. The guideline published by WHO for the application of convalescent whole blood and plasma in the treatment of EVD indicates that patients with confirmed infection in the early stage of the disease should be considered for receiving convalescent blood or plasma [20]. In addition, it is stated that venous blood samples should be taken from the patient and collected in tubes with or without anticoagulant (to obtain plasma and serum) in volumes of 5 mL. The plasma sample is used for ABO and RhD blood typing, while the serum sample is used to measure the level of viral load before receiving the treatment (establishing a baseline). It is also stated that another serum sample should be taken a day after the transfusion to measure the viral load after the treatment. The guideline also states that two other serum samples should be collected from the recovered patients before discharge on two consecutive days. Regarding the selection of whole blood and plasma units, it is stated that ABO and RhD groups should match for both whole blood and plasma transfusion. If ABO groups of the patient and donor match, the guideline states that cross-matching tests may be omitted to reduce the risk of handling infectious blood samples. In desperate situations, it is stated that nonmatching RhD group units may be used, but extra caution should be given for RhD-negative women of child-bearing age. In addition, when it is not possible to determine the blood group of patients, Group O convalescent whole blood and Group AB convalescent plasma could be administered.

The guideline published by WHO for the application of convalescent whole blood and plasma in the treatment of EVD indicates that patients with confirmed infection in the early stage of the disease should be considered for receiving convalescent blood or plasma [20]. The administration of convalescent whole blood and plasma should be performed according to the standard procedure for transfusion. The WHO guideline states that one unit of convalescent whole blood, which is collected in 350/450 mL blood bags, should be transfused to an adult patient. Regarding convalescent plasma, the guideline proposes that 400–500 mL of convalescent plasma in two doses of 200–250 mL that are collected from two different donors should be administered to adult patients. In addition, the guideline states that convalescent whole blood or plasma should be administered to minors at a dose of 10 mL/kg could be considered. Careful monitoring of the patient, especially in the first 20 min, is advised. The slow intravenous transfusion should be completed within 1–4 h. Another point made in the WHO guideline is that the patients should receive a full treatment course with an effective antimalarial drug in areas with high malaria transmission. Finally, it is stated that the call for receiving another treatment with convalescent blood or plasma should be made based on the response observed after the treatment and on the level of Ebola virus-neutralizing antibodies in the donor and the patient [20].

The guideline published by the FDA during the outbreak of COVID-19 is another useful source to establish selection criteria for the administration of convalescent plasma [29, 31]. Initially, the FDA authorized the emergency use of convalescent plasma in the treatment of hospitalized COVID-19 patients (August 23, 2020) [31]. The EUA was later limited for both donor and patient selection criteria. Regarding donors, only those who have a high titer of antibodies were qualified to donate convalescent plasma. In addition, only the patients with an impaired immune system were found eligible to receive the convalescent plasma in both inpatient and outpatient settings.  Table 3 provides a recommendation matrix for different clinical scenarios in convalescent plasma therapy of COVID-19 patients [43]. The FDA also encourages more clinical trials under IND to answer the questions regarding the efficacy of treatment for different cohorts.  Table 4 lists the major guidelines for convalescent plasma transfusion.

## 4. Manufacture of hIVIGs From Convalescent Plasma

### 4.1. Preparation of Pooled Convalescent Plasma

In order to perform plasma fractionation, it is necessary that thousands of plasma donations be pooled. The WHO guideline for the preparation of human plasma for fractionation is a useful document in this matter [44]. The viral screening tests employed in the donor selection step should be used for the pooled plasma to ensure the safety of plasma-derived products. The USP has a comprehensive section (1240) that discusses virus testing of plasma intended for manufacturing [25]. A useful strategy to avoid the loss of large amounts of plasma is minipooling. Plasma units can be pooled in smaller volumes, and screening tests can be performed on these minipools. It is noteworthy that not all tests can be performed on these minipools. In fact, the USP clearly states that NATs can be performed on these minipools to detect viral load due to their high sensitivity. On the other hand, serological tests (either antibody or antigen) must be performed on each individual plasma unit, as combining the plasma into a pool reduces the viral load to a point below their detection limit. The donor selection criteria are nearly the same as those required for transfusion. The WHO guideline proposes a strict implementation of GMP in the collection of a high-scale plasma pool [44]. The USP requires manufacturers to follow current GMP regulations based on federal CFRs and FDA guidelines. Twenty-one CFR § 600 and 21 CFR § 606 are two of the sources mentioned in the USP, which encompass instructions intended for general biological products and blood-related products, respectively. An additional point emphasized is regarding the traceability of units collected and the final product obtained from those units. This is especially important to be able to trace back the source of possible problems and ensure the safety of the final products obtained.

The plasma for fractionation is obtained either from whole-blood donations (recovered plasma) or through apheresis (source plasma). Apheresis is a technique that allows the separation of soluble components of blood from cells, which, in this case, are transfused back into the donor's bloodstream. According to the USP, plasma collected by the apheresis method is the main source of plasma for the purpose of fractionation [25]. The WHO guideline states that plasma is better to be obtained through apheresis as it offers a number of advantages [44]. First and foremost, a higher volume of plasma can be obtained through apheresis (450–800 mL, as compared to 100–260 mL obtained from whole-blood donations). Second, some proteins (like labile coagulation factors) have a higher content in apheresis plasma. Lastly, regarding convalescent plasma, as we are dealing with recovered patients, it would be much safer if their blood cells were to be transfused back into the donors to reduce the physiological burden. For the optimal recovery of labile proteins, plasma is better to be frozen rapidly within 24 h of its collection to obtain a plasma unit core temperature of −25°C after 12 h of placing it in the freezing apparatus [45]. However, immunoglobulins (IGs) are fairly stable components of plasma, and according to European Pharmacopeia, when nonlabile proteins are intended, plasma obtained by plasmapheresis should be frozen in a chamber of −20°C or below within 24 h of collection. For recovered plasma (obtained from whole-blood), the plasma should be separated from cellular components and be frozen in a chamber of −20°C or below within 72 h of whole-blood donation [45]. The product obtained through any of the abovementioned conditions is referred to as fresh frozen plasma (FFP). European Pharmacopeia states that the FFP should be kept at −20°C or below at all times during the storage. However, the FFP unit can still be used if the temperature on no occasion has gone up −5°C, the temperature has not gone above −15°C more than one occasion, and lastly, the total time the FFP unit temperature was above −20°C is not more than 72 h. The first homogenous pooled plasma (after the removal of cryoprecipitate) can be analyzed with regard to viral infections, and additionally, the intended neutralizing antibody titer (EMEA/CHMP/BWP/3794/03 and EMEA/CHMP/BWP/298388/05) [45]. According to the USP, the level of viral load in the pooled plasma should be shown to be lower than the capacity of the viral reduction process [25]. Inventory hold is another strategy commonly used to ensure the safety of plasma-derived products in which the plasma units are stored for 60 days before pooling. The idea behind the inventory hold protocol is that it allows the donors negative for certain infections to produce a detectable titer of antibody [25]. Despite the efforts made in donor selection and screening, this viral-reduction step is considered crucial, and great care should be given to its validation, as the incidents of viral transmission have occurred in the past [46].

### 4.2. Purification of IGs From Donated Plasma Pool

Table 5 lists the major guidelines for hIVIG manufacture. Oftentimes, cryoprecipitation is employed as the first step of the purification process [46]. Different physical-, chemical-, and chromatography-based methodologies could be taken into consideration based on available facilities; however, alternative methods of manufacture for the preparation of medicinal products are rarely accepted by authorities [46]. While chromatography-based purification strategies provide a more efficient separation experience, chemical precipitation methods are more straightforward and time-saving procedures. Both approaches could be adapted for IG production with high purity and acceptable recovery rate; however, ethanol fractionation, developed by Cohn et al. in the 1940s, is the traditional approach for the purification of albumin and IGs [46–48]. The procedure consists of different cold ethanol precipitation applied through several steps. Each of the steps leads to products that should meet specific requirements. The correct implementation of the precipitation procedure will even reduce the viral load in the final product. The European Medicines Agency suggests that clear specifications for ethanol and protein concentration, temperature, pH, and ionic strength, time of each treatment, and acceptable degree of tolerance for the final products be established. The guideline also calls for providing similar data and specifications if using methods based on the chemicals. The use of methods based on caprylic acid is shown to have an effect on viral load reduction. The chromatographic methods have also been used to manufacture plasma-derived products. According to the EMA guideline on plasma-derived medicinal products, the efficacy of chromatographic methods highly depends on factors like column capacity, concentration of protein, ionic strength, pH, contact time, flow rate, and temperature. Parameters and factors such as the storage condition of the column, preservation, sanitation, and methods of regeneration should be described. In order to ensure the safety of plasma-derived products, routine viral inactivation procedures must be performed before, after, or in conjunction with the purification process according to the criteriet al. a of European Pharmacopeia and other relevant documents [46]. The viruses pose diverse physicochemical properties, and therefore, the guidelines suggest incorporating at least two different and complementary viral inactivation/removal steps to ensure the safety of the final product. According to the EMA guideline on plasma-derived medicinal products, removal or inactivation of all known nonenveloped viruses through a single step is hard to achieve, and therefore, one of the virus reduction treatments should be especially effective against these viruses [46]. That said, the guideline also states that if a single viral/inactivation is shown to be both reliable and effective against all types of viruses, a second step might not be necessary. Viruses can be divided into those that are either susceptible or resistant to virus inactivation/removal methods. The characteristics of each viral strain should be taken into consideration in the design of virus reduction steps. For instance, nonenveloped viruses are resistant to heat inactivation treatment, while small viruses like circoviruses are immune to filtration techniques (which will penetrate the membrane). According to the EMA guideline on plasma-derived medicinal products, the effectiveness of viral reduction steps against all types of viruses also decreases the chance of an unknown or emerging viral infection. This point should be given special attention when manufacturing plasma-derived products from recovered patients of an emerging viral outbreak. The EMA guideline on plasma-derived medicinal products mentions some of the methods that contribute to viral reduction and their common limitations [46]. These methods include ethanol precipitation, heating (both in aqueous solution and lyophilized form), some solvent/detergent treatments, filtration techniques, and reduction of pH. Caprylic acid precipitation is a well-known and simple procedure for IG purification from convalescent plasma. Caprylic acid has shown a great capacity to remove enveloped viruses [49]. In addition to its virus removal capacity, caprylic acid could be used to deplete plasma from non-IG proteins [50, 51]. The impurities in this procedure mainly include high molecular weight plasma proteins, IG polymers, and protein aggregates. To remove IG polymers and protein aggregates, the supernatant from caprylic acid precipitation could be subjected to a PEG precipitation step. Looking at the literature, PEG precipitation has been reported to remove not only protein polymers and aggregates but also to an efficient virus removal procedure. This removal step also enables the downstream nanofiltration of caprylic acid supernatant for highly efficient removal of nonenveloped viruses [52]. In many industrial IG separation methodologies, a combination of chemical- and chromatography-based purification strategies is used. For example, a downstream ion-exchange chromatography purification on caprylic acid supernatant could result in a purer product. Also, this chromatography process is a perfect step for the removal of residual PEG [53, 54]. The purification procedure could be adapted from existing protocols and regulations, which are licensed for the manufacture of normal human immunoglobulin for intravenous (IVIG) administration.

Validation of effectiveness for viral reduction steps is as crucial and cumbersome as the process itself. Among all biological products, those that are produced from undefined sources and species closely related to humans, such as human blood, pose the greatest danger of transmitting a viral infection. Therefore, the validation of the virus reduction step should be given special attention. There are guidelines specifically provided to aid the establishment of an appropriate validation method (CPMP/BWP/268/95) [55].

According to the guideline, the aim of the validation process is to ensure that the viral reduction step will definitely remove or inactivate all known viruses and also will reduce the chance of transmitting a novel virus [55]. The assessment is done by deliberating on introducing specific viruses to different steps of manufacturing and showing how much each step is effective in reducing the viral load. The viruses chosen for the purpose of assessment should have two characteristics: First, they should be similar to those that are most likely to be present in human plasma and cause an infection. Second, they should represent a wide range of viruses regarding their physicochemical properties. According to this, it is advisable to include a virus of the same class for validation of the IVIG manufacturing process of a previously unknown viral outbreak. It should also be mentioned that viral strains grown in a lab might show a high degree of difference from the native strains and other laboratory strains of the same virus. The important point is that the process being investigated causes a reproducible reduction of viral load. In addition, the key parameters influencing the efficacy of viral reduction should be determined, for example, time of treatment, temperature, and so forth, and the kinetic process should be investigated. In addition, it should be determined whether the cause of virus reduction is the removal of viruses or their inactivation. In studying the kinetics of virus reduction, the degree of assurance for the correct implementation of the method and its efficacy should be high. For instance, a process shown to remove/inactivate viruses rapidly at the beginning of treatment (at the beginning of the incubation time) has a higher assurance as compared to the one that does so during the end of treatment [55]. In addition, the GMP prevents manufacturers from introducing a virus to any of the equipment used for the production of biological products. In fact, the guideline states that the validation study should be done in another facility specifically designed for scaling down the manufacturing process with relevant equipment and personnel [55]. Besides the efficacy of treatments in the reduction of viral load, the effect of each step, both alone and in combination with other procedures, on the integrity of the final product should be investigated [46]. For instance, the formation of neoantigens, increased thrombogenicity, and residues of toxic chemicals used are all possible outcomes of the implementation of viral reduction steps [46]. Taken together, it is obvious that the validation process is an estimation rather than a definite measure of how much the process is effective for reducing the viral load.

### 4.3. Analytical Studies and Quality Controls

The Committee for Proprietary Medicinal Products (CPMP) has some insightful material regarding the quality control of plasma-derived products. Regarding in-process control, the guideline states that the manufacturer should describe all the procedures for production, monitoring of equipment, details of control steps, means of sampling and the storage of samples, and testing protocols. In addition, it states that great care should be taken when pooling the plasma units to avoid the accidental introduction of any foreign material and contamination [46]. The guideline also suggests that critical parameters, such as pH, temperature, ethanol concentration, bacterial count, level of endotoxins, and so forth, should be monitored and documented fittingly. For the identification of appropriate stages to implement in-process controls and setting limitations for parameters (action limits), the guideline references CPMP/ICH/365/96 [46]. In that reference, it is stated that the in-process controls should be set at critical decision-making steps and where the data can be used to assess the accuracy of the production process. The data obtained from in-process assessments can be used to establish action limits and gradually refine acceptance criteria as the manufacturer gains experience [56]. CPMP states that all plasma-derived products must meet the specifications outlined in appropriate European Pharmacopeia monographs. Each product must possess specific characteristics. There are various sections in European Pharmacopeia describing the requirements for the preparation of normal human IGs (for intramuscular and subcutaneous injections), normal human IGs for intravascular injections (IVIG), and several other specific sections for IGs prepared from immunized subjects (against measles, hepatitis, etc.). The antibody purified from immunized subjects should conform to the criteria for preparing normal human IGs except for the minimum number of plasma units required and the minimum total protein concentration.

The manufacture of hIVIG from convalescent plasma during an emerging viral outbreak for IV injection should be performed under regulations established to produce normal human IVIG injection. These requirements ensure the final product's viral/bacterial safety and physicochemical characteristics. The virus removal efficiency of the purification procedure is to be evaluated by determining the load of enveloped and nonenveloped viruses. The details of the viral reduction process and its validation were discussed in the previous section (purification process). The final product should be negative for hepatitis B and C surface antigens as well as HIV-1 p24 antigen and anti-HIV antibodies. The residual chemical content from purification and virus-inactivating procedures should be quantified using gas chromatography or HPLC. The concentration of IGs should be at least three times higher than that of the initial pool. Moreover, the final product should define the distribution of IgG (G class of the IG). In addition, the function of IG Fc should be tested according to the methods described in European Pharmacopeia (2.7.9) [45]. The product can be prepared in freeze-dried form (dissolved within 30 min at 20°C–25°C) or as a stabilized solution. In order to determine the total protein content of the final product, a method of nitrogen determination based on sulfuric acid digestion must be implemented according to European Pharmacopeia (2.5.9). The minimum protein concentration should be 30 g/L and between 90% and 110% of the amount stated on the product label. The protein components of the product are advised to be investigated by cellulose acetate or agarose electrophoresis, methods which are described in detail under the title zone electrophoresis (2.2.31) in European Pharmacopeia. The identification of IGs is suggested to be confirmed by an immunoelectrophoresis technique using human antiserum. The product in question and normal human serum are applied as a sample and control at a concentration of 10 g/L. Only small amounts of other protein may be present in the final product. Other necessary analytical studies include the product's protein content, which is to be determined using liquid chromatography with an appropriate stationary phase to determine the molecular weight distribution of the product. The content of IG polymers and aggregates should be less than 3% of the total IG content of the product (total chromatography area) when quantified by analyzing the chromatogram EP [57]. The stationary phase used for the liquid chromatography should be able to separate the proteins within the range of 10–500 kDa. For this purpose, the European Pharmacopeia suggests hydrophilic silica gel with specifications that provide efficient separation in the abovementioned molecular weight range. The retention time of the product (IgG) should correspond to that of the reference material with a margin of error of 2%. In addition, the sum of peaks related to IgG (in its monomer and dimer form) should not be less than 90% of the total area of the chromatogram. Analytical studies should be designed and performed under pharmacopeia monographs or other local regulations. In addition, a core summary of product characteristics (SPC) must be provided for the final product, which summarizes the product characteristics to regulators.

### 4.4. Administration

The administration of hIVIG during an emerging viral outbreak is most likely arbitrary due to the lack of relevant clinical data. The decisions on the indications and dose of administration remain to be taken by local health authorities and clinical committees. A preclinical study must be implemented for the evaluation of product safety. The efficacy of the product should be evaluated through randomized clinical trials. Referring to the literature, Hung et al. have used 0.4 g/kg purified hIVIG against H1N1 influenza in a randomized clinical trial [58]. Another study by Davey et al. evaluated the efficacy of 0.25 g/kg purified anti-influenza hIVIG against influenza A and B [59]. For anti-COVID-19 hIVIG, a number of clinical trials have used a single dose of hIVIG at concentrations ranging from 0.15 to 0.3 gr/kg [60–62]. In another trial, anti-SARS-CoV-2 IGs were given at a dose of 2 gr/kg over 4 or 5 days by infusions of 0.5 and 0.4 gr/kg, respectively [63]. Commonly, the existing settings and guidelines for administering IVIG could be reviewed and adapted for administering hIVIG.

## 5. Conclusion

It should be noted that convalescent plasma and hIVIG in treating infectious diseases have their own advantages or disadvantages. Plasma transfusion is technically simpler and rapidly applicable. Moreover, the albumin content of the whole plasma theoretically could contribute to the compensation of protein loss and correction of oncotic pressure in critically ill patients. On the other hand, the infusion volume in plasma transfusion is as high as 500 mL, which may not be tolerated by a critically ill patient or result in detrimental effects. Contrarily, hIVIG provides much more concentrated antibodies in considerably lower injection volume. In addition, hIVIG administration does not need an ABO blood group or rhesus antigen match between the donor's and recipient's blood. More importantly, hIVIG manufacturing procedures include virus removal steps, and the final product undergoes analytical studies and quality control to ensure maximum safety and quality. Finally, the IG amount could be easily controlled, which is not feasible for plasma transfusion. Therefore, local health authorities should decide on a more applicable approach regarding the local status of disease outbreaks and available facilities.

Special attention should be paid to the fact that the existing clinical data on the effectiveness of both convalescent plasma and purified hIVIGs against viral infections are controversial (Table 6). Due to numerous challenges in conducting large-scale randomized clinical trials, convalescent plasma and hIVIG remain empirical therapeutic options. As mentioned before, it is unlikely that any reliable clinical data on the efficacy of convalescent products will be available during an emerging viral outbreak. Thus, the present proposal should be considered a research roadmap for developing a therapeutic approach. The decision on high-scale production of hIVIG for public administration is to be taken by health authorities based on data obtained from randomized clinical trial studies.

## Figures and Tables

**Table 1:** Advantages and limitations of using convalescent plasma and hIVIG in the treatment of viral infections.

	**Convalescent plasma**	**Hyperimmune globulins**
Advantages	• A feasible choice in desperate conditions • Easier to set-up • Much more economical	• Can be administered with relative ease • High biological safety standards • Approximately 10-fold more concentrated than convalescent plasma

Limitations	• A higher risk of viral transmission • Risk of anaphylactic shock	• Requires a significant amount of infrastructure in place • Takes a long time to establish, especially during crisis • No enough evidence for certainty of effectiveness

**Table 2:** The major guidelines for donor selection.

	**Guidelines and settings**
General and specific donor selection	• Red Cross, “Criteria for Blood Donor Selection” • WHO, *Blood Donor Selection: Guidelines on Assessing Donor Suitability for Blood Donation* • American Association of Blood Banks (AABB) standards and guidelines • Code of federal regulations: CFR 640.31 (§ CFR 630.10, § CFR 630.15, § CFR 630.30, and § CFR 610.41.) • FDA, “Investigational COVID-19 Convalescent Plasma: Guidance for Industry” • WHO interim guidance for the use of convalescent whole blood or plasma collected from patients recovered from Ebola virus

Diagnostic methods for detecting the presence of an active viral infection	• United States Pharmacopeia (USP) and national formulary (NF) • Clinical and Laboratory Standards Institute (CLSI) guidelines for qualitative and quantitative tests

**Table 3:** Convalescent plasma therapy for different clinical scenarios in COVID-19 patients.

**Clinical scenario**	**Recommendation**	**Effectiveness certainty**
Immunocompetent patients hospitalized with COVID-19	Strong recommendation against	Moderate
Immunocompetent patients hospitalized with COVID-19	Conditional usage specially in patients who do not qualify for other treatments	Very low
Ambulatory patients with mild-to-moderate viral disease	Conditional use for patients who are at risk of progression to severe disease who qualify for no other treatment option	Low

**Table 4:** The major guidelines for the urgent initiation of plasma transfusion from recovered patients to critically ill patients.

	**Guidelines**
General plasma transfusion considerations	• WHO, “Clinical Transfusion Practice Guidelines for Medical Interns” • AABB, “Evidence-Based Practice Guidelines for Plasma Transfusion”

Establishment of patient inclusion and exclusion criteria for receiving convalescent plasma	• FDA, “Investigational COVID-19 Convalescent Plasma: Guidance for Industry” • “Use of Convalescent Whole Blood or Plasma Collected From Patients Recovered From Ebola Virus Disease for Transfusion, as an Empirical Treatment during Outbreaks”

**Table 5:** An overview of the manufacture and use of hyperimmune globulins in the treatment of emerging viruses. All criteria and regulations must be reviewed and agreed to by local health authorities and regulators.

**Procedure**		**Guidelines and settings**
Manufacture of hyperimmune globulins	Preparation of pooled convalescent plasma	• WHO Technical Report, Series No. 941, 2007, Annex 4, “Recommendations for the Production, Control and Regulation of Human Plasma for Fractionation” • European Pharmacopeia monograph: Human plasma for fractionation, 2020:0853 • European medicines agency guideline on plasma derived medicinal products (current effective version: EMA/CHMP/BWP/706271/2010)
	Purification of IGs from donated plasma pool	• Adaptation from licensed procedures for IVIG manufacture • European medicines agency guideline on plasma derived medicinal products (current effective version: EMA/CHMP/BWP/706271/2010)
	Analytical studies and quality controls	• Russian Pharmacopeia monograph: Normal human immunoglobulin for intravenous administration, PM.3.3.2.0008.15 • European medicines agency, committee for medical products for human use (CHMP): Core SPC for human normal immunoglobulin for intravenous administration (IVIGs)

Administration of hyperimmune globulins	General criteria for IVIG administration	• NHS, “Intravenous Immunoglobulin (IVIg) Prescribing Guidance”
	Specific criteria for the administration of hyperimmune globulins	• Establishment of administration guidelines by clinical committees

**Table 6:** Outcomes of major clinical trials of convalescent plasma and hIVIG in viral outbreaks.

**Disease**	**Main findings**	**Ref.**
COVID-19	The data from RECOVERY and PlasmAr trials suggest that convalescent plasma does not benefit patients in most settings. Better outcomes are associated with immunocompromised patients. hIVIG may improve outcome for immunocompromised patients.	[64–70]
MERS	Trials of convalescent plasma were limited and unconvincing. Some small studies suggested no beneficial effect.	[71, 72]
Ebola virus disease (EVD)	Trials were limited in size. The use of convalescent plasma did not improve survival chances significantly.	[21, 73–75]
Lassa fever	Lack of randomized clinical trials. Conflicting results from small studies.	[76–78]
Influenza	Only limited overall efficacy for both convalescent plasma and hIVIG.	[59, 79–81]
Argentine hemorrhagic fever	Results from convalescent plasma trials suggest a significantly reduced mortality.	[82–85]

## Data Availability

Data sharing is not applicable to this article as no datasets were generated or analyzed during the current study.
